# Synthesis, Characterization, and Potential Application of Cyclodextrin-Based Polyrotaxanes for Reinforced Atelocollagen Threads

**DOI:** 10.3390/polym15153325

**Published:** 2023-08-07

**Authors:** Riku Kubota, Ichiro Fujimoto

**Affiliations:** Koken Research Institute, Koken Co., Ltd., 1-18-36 Takarada, Tsuruoka-shi, Yamagata 997-0011, Japan

**Keywords:** atelocollagen thread, crosslinking, cyclodextrins, polyrotaxane, slide-ring features

## Abstract

Preparing strong and flexible atelocollagen-based materials for biomedical applications is still a challenging task. To address this challenge, this study describes the synthesis and characterization of water-soluble polyrotaxanes (PRs) with different coverage ratios and molecular weights of axle polymers, and their potential applications for PR-reinforced atelocollagen threads (PRATs). A novel method was established for the syntheses of PRs with relatively low coverage ratio at the sub-gram scale, in which the aldehyde groups were employed as crosslinking sites for preparing the PRATs via reductive amination. The aldehyde groups were successfully quantified by ^1^H nuclear magnetic resonance spectroscopy using 1,1-dimethylhydrazine as an aldehyde marker. Fourier-transform infrared and thermogravimetric analysis measurements supported the characterization of the PRs. Interestingly, tensile testing demonstrated that coverage ratio affected the mechanical properties of the PRATs more strongly than molecular weight. The insights obtained in this study would facilitate the development of soft materials based on atelocollagens and PRs.

## 1. Introduction

Polyrotaxanes (PRs) are essential components of composite materials [1,2,3,4,5,6]. Since the discovery of the first PR in 1992, various types of macrocyclic compounds and polymers have been evaluated in the formation of supramolecular architectures [1,2,3,4,5,6]. Currently, PR consisting of cyclodextrin (CD) and/or an amphiphilic polymer (e.g., polyethylene glycol (Peg)) is regarded as one of the promising slide-ring materials for various functionalities [7,8,9,10,11]. Multiple attempts were made to apply PRs as therapeutic and diagnostic tools in the field of biomedical chemistry [12,13,14,15,16,17]. For example, PRs are utilized for crosslinking with biomacromolecules, delivery of bioactive molecules, and surface modification of cell-adhesive materials [18,19,20,21,22,23], which indicates that selecting proper design and synthetic methods is important for implementing their desired functionalities as biomaterials. However, some of the previously used synthetic approaches for PR crosslinking remain controversial [24,25,26,27,28,29,30].

Collagen is a natural protein and major component of mammalian organs and tissues [31,32]. Owing to the abundance and biological benefits of collagens, collagen-based biomaterials have been widely employed in various fields [33,34,35,36]. Pepsin digestion produces an atelocollagen lacking N- and C-terminal telopeptides, which reduces the potential immunogenicity of collagen [37,38]. Atelocollagens are promising materials utilized together with collagens in advanced therapeutics [39,40,41]. We have previously discussed the benefits of applying atelocollagens in oligonucleotide therapeutics, drug development, and regenerative medicine [23,42,43,44]. However, the preparation of flexible and strong atelocollagens remains a challenging task despite their potential applicability in human treatment procedures (e.g., ligament and tendon repair).

**Figure 1:**
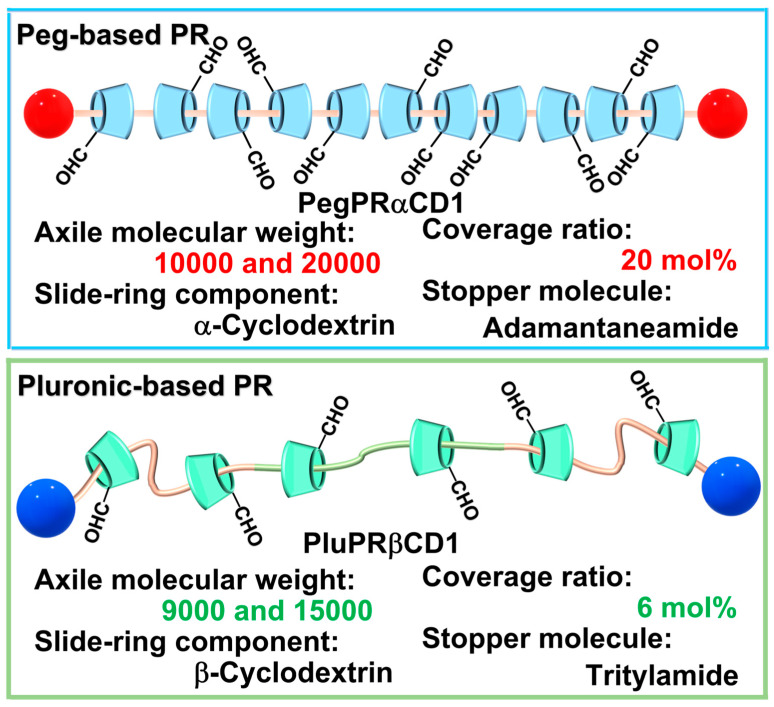
Schematic illustrations of PRs for PRATs.

Herein, we report the synthesis, characterization, and potential applications of CD-threaded PRs for reinforced atelocollagen threads. Two different types of PRs were synthesized and crosslinked to investigate the effects of their molecular weight and coverage ratio on the mechanical properties of the PR-reinforced atelocollagen threads (PRATs) (Figure 1).

## 2. Materials and Methods

### 2.1. Chemicals

**Scheme 1:**
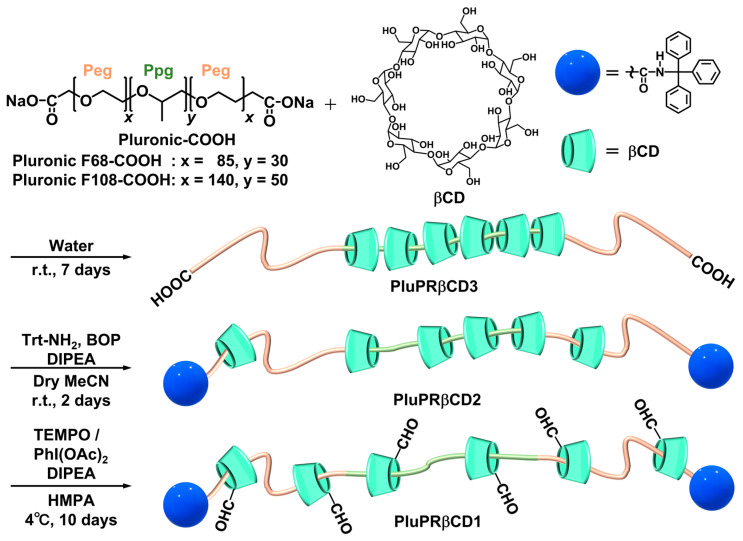
Synthetic scheme for PluPRβCD1.

Hydroxyl group-terminated polyethylene glycols (Peg_10k_–OH and Peg_20k_–OH), (benzotriazol-1-yloxy)tris(dimethylamino)phosphonium hexafluorophosphate (BOP), and βCD were purchased from FUJIFILM Wako Pure Chemical Corporation (Osaka, Japan). αCD, iodobenzene diacetate (PhI(OAc)_2_), *N*,*N*-dimethylformamide (DMF), dichloromethane, diethyl ether, dry acetonitrile (MeCN), and 2,2,6,6-tetramethylpiperidine 1-oxyl (TEMPO) were procured from Kanto Chemical Co., Inc (Tokyo, Japan). DMF was distilled under reduced pressure and stored with a molecular sieve prior to use. CD monoaldehydes (i.e., αCD-CHO and βCD-CHO) were synthesized according to the previous method [23]. *N*,*N*-diisopropylethylamine (DIPEA), hexamethylphosphoric triamide (HMPA), triphenylmethylamine (Trt–NH_2_), [bis(trifluoroacetoxy)iodo]benzene (PhI(OAcTf)_2_), 1,1-dimethylhydrazine (DMHZ), and sodium cyanoborohydride (NaBH_3_CN) were obtained from Tokyo Chemical Industry Co., Ltd (Tokyo, Japan). Amino group-terminated Pegs (Peg_10k_–NH_2_ and Peg_20k_–NH_2_) were synthesized from Peg_10k_–OH and Peg_20k_-OH, respectively, via a typical Gabriel amine synthesis procedure described elsewhere [8]. PRs with a coverage ratio of 20 mol% were synthesized using Peg and αCD (Peg_10k_PRαCD1 and Peg_20k_PRαCD1). αCD-threaded *pseudo*-PR (PegPRαCD3) and adamantane end-capped PR (PegPRαCD2) were prepared according to a previously developed method [45]. To produce PRs with lower coverage ratios, triblock copolymer Pluronic and βCD were employed as shown in Scheme 1 (Plu_9k_PRβCD1 and Plu_15k_PRβCD1). Hydroxyl group-terminated Pluronic reagents (Plu_9k_–OH and Plu_15k_–OH) were purchased from Sigma–Aldrich (St. Louis, MO, USA). Carboxyl group-terminated Pluronic compounds (Plu_9k_–COOH and Plu_15k_–COOH) were synthesized via bleach oxidation as described previously [46]. A Spectra/Por dialysis membrane (MWCO: 1 kDa) was used for purification in each step.

### 2.2. PegPRαCD1 General Synthesis Procedure

**Scheme 2:**
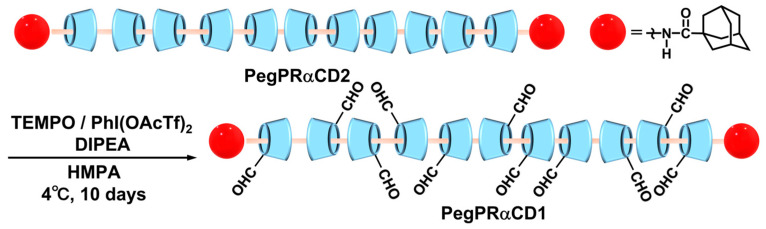
Synthetic scheme for PegPRαCD1.

Synthesis of PegPRαCD1 from PegPRαCD2 is described in Scheme 2. PegPRαCD2 (0.1 g) and DIPEA (0.2 mL) were dissolved in HMPA (3 mL) and cooled to 4 °C. PhI(OAcTf)_2_ (36 mg, 0.084 mmol) and a catalytic amount of TEMPO (3.9 mg, 0.025 mmol) were added to the obtained solution. The resulting mixture was stirred at 4 °C for 10 days and then added dropwise to diethyl ether. The obtained precipitate was repeatedly centrifuged in cold MeCN and dialyzed in deionized water. The dialyzed aqueous solution was freeze-dried to produce Peg_10k_PRαCD1 or Peg_20k_PRαCD1 with a nearly quantitative yield. IR (KBr) *ṽ* = 3377, 2911, 1645, 1152, 1082, and 1032 cm^−1^. See Section 2.4 regarding the aldehyde marking with DMHZ for ^1^H NMR measurement.

### 2.3. Synthesis of PluPRβCD1

#### 2.3.1. PluPRβCD3 General Synthesis Procedure

Plu–COOH (1 g) and βCD (1 g, 0.88 mmol) were dissolved in deionized water (56 mL) and stirred at 25 °C for 7 days. A white precipitate was gradually formed during the reaction, which was thoroughly centrifuged (14,000× *g*, 20 min) and washed with cold water. Freeze drying the resulting precipitate produced PluPRβCD3 as a white powder. The further addition of βCD (0.36 g) to the supernatant and repetition of the above-mentioned procedure generated more PluPRβCD3. In total, approximately 0.7 g of Plu_9k_PRβCD3 and Plu_15k_PRβCD3 was obtained from one gram of Plu–COOH. δ_H_ (DMSO-d_6_): 1.01–1.05 (m, 3H, C*_j_*-H), 3.30–3.38 (m, C*_b_*_,*d*_-H and C*_k_*_,*l*_-H), 3.41–3.67 (m, 32H, C*_c_*_,*e*,*f*_-H and C*_m_*-H), 3.94 (br, 4H, C*_n_*-H), 4.37–4.40 (m, 7H, OH*_g_*), 4.83–4.84 (m, 7H, C*_a_*-H), 5.63–5.68 (m, 14H, OH*_h_*_,*i*_) (Figure S2).

#### 2.3.2. PluPRβCD2 General Synthesis Procedure

BOP (0.34 g, 0.76 mmol), Trt–NH_2_ (0.2 g, 0.76 mmol), and DIPEA (0.14 mL, 0.82 mmol) were dissolved in dry MeCN (3 mL). Powdered PluPRCD3 (0.5 g) was added to the resulting solution under vigorous stirring. The obtained reaction mixture was stirred at 25 °C for 2 days. Subsequently, it was repeatedly centrifuged (1500× *g*, 20 min) in cold MeCN until the supernatant became colorless. The precipitate was thoroughly washed with dichloromethane and filtered. The dialysis of the solid residue followed by freeze drying produced PluPRβCD2 as a white powder. The yields of Plu_9k_PRβCD2 and Plu_15k_PRβCD2 were 0.24 g (48%) and 0.20 g (40%), respectively. δ_H_ (DMSO-d_6_): 1.03–1.05 (br, 3H, C*_j_*-H), 3.28–3.38 (br, C*_b_*_,*d*_-H and C*_k_*_,*l*_-H), 3.42–3.66 (br, 32H, C*_c_*_,*e*,*f*_-H, C*_m_*_,*n*_-H), 4.45–4.47 (m, 7H, OH*_g_*), 4.82–4.83 (m, 7H, C*_a_*-H), 5.68–5.75 (m, 14H, OH*_h_*_,*i*_), 7.16–7.32 (m, trityl group) (Figure S3). IR (KBr) *ṽ* = 3362, 2905, 1649, 1367, 1157, 1082, and 1030 cm^−1^.

#### 2.3.3. PluPRβCD1 General Synthesis Procedure

PluPRβCD2 (0.12 g) was dissolved in HMPA (3 mL) and cooled to 4 °C. PhI(OAc)_2_ (22 mg, 0.065 mmol) and a catalytic amount of TEMPO (3.3 mg, 0.0195 mmol) were added to the prepared solution, and the resulting mixture was stirred at 4 °C for 10 days. The reaction mixture was added dropwise to diethyl ether, and the obtained precipitate was centrifuged (1500× *g*, 20 min). Subsequently, the precipitate was washed and centrifuged using cold MeCN and dialyzed in deionized water. Freeze drying the inner dialysis solution produced Plu_9k_PRβCD1 or Plu_15k_PRβCD1 with a nearly quantitative yield. IR (KBr) *ṽ* = 3389, 2934, 1649, 1456, 1155, 1082, and 1030 cm^−1^. See the section below regarding the aldehyde marking with DMHZ for ^1^H NMR measurement.

### 2.4. Quantification of Aldehyde Groups Using DMHZ

**Figure 2:**
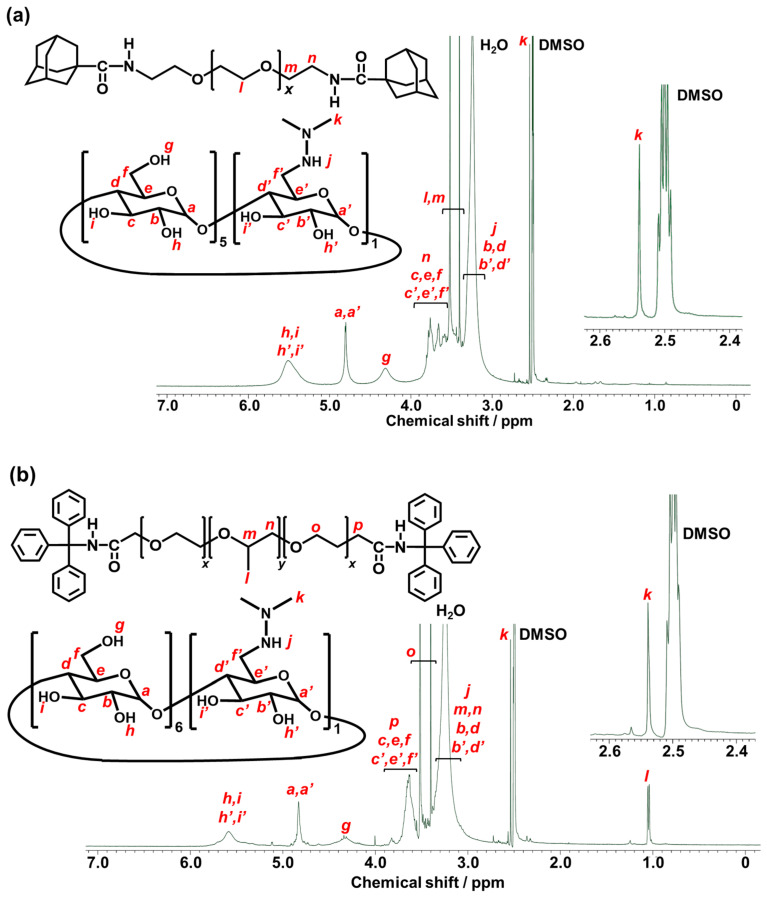
^1^H NMR spectra of (**a**) Peg_10k_PRαCD1-DMHZ and (**b**) Plu_9k_PRβCD1-DMHZ (400 MHz, 318 K, DMSO-*d*_6_).

PegPRαCD1 or PluPRβCD1 (10 mg) was dissolved in DMHZ-buffered water (0.2 M, pH = 8.2) and stirred at 37 °C for 48 h. NaBH_3_CN was added to the obtained solution at a final concentration of 0.2 M. The resulting mixture was stirred at 37 °C for another 48 h and subsequently dialyzed in deionized water for 24 h. Freeze drying the inner dialysis solution and thoroughly washing with ethanol produced the DMHZ-labeled PegPRαCD1 or PluPRβCD1 with a nearly quantitative yield. For PegPRαCD1-DMHZ δ_H_ (DMSO-d_6_): 2.54 (s, 3H, C*_k_*-H), 3.24 (br, N*_j_*-H, C*_b_*_,*b*’,*d*,*d*’_-H), 3.40–3.52 (m, 4H, C*_l_*_,*m*_-H), 3.59–3.81 (br, 28H, C*_n_*-H, C*_c_*_,*e*,*f*_-H and C*_c_*_’,*e*’,*f*’_-H), 4.31 (br, 6H, OH*_g_*), 4.80–4.81 (br, 6H, C*_a_*_,*a*’_-H), 5.51 (br, 6H, OH*_h_*_,*h*’,*i*,*i*’_) (Figure 2a).

For PluPRβCD1-DMHZ δ_H_ (DMSO-d_6_): 1.04–1.05 (br, 3H, C*_l_*-H), 2.54 (s, 3H, C*_k_*-H), 3.25 (br, N*_j_*-H, C*_b_*_,*b*’,*c*,*c*’_-H and C*_m_*_,*n*_-H), 3.40–3.52 (m, 4H, C*_o_*-H), 3.64–3.82 (br, 32H, C*_p_*-H, C*_c_*_,*e*,*f*_-H and C*_c_*_’,*e*’,*f*’_-H), 4.34 (br, 7H, OH*_g_*), 4.83 (br, 7H, C*_a_*_,*a*’_-H), 5.59 (br, 14H, OH*_h_*_,*h*’,*i*,*i*’_) (Figure 2b).

### 2.5. Preparation of PRATs

PRATs were fabricated according to a procedure described in our previous study [23]. Briefly, an atelocollagen solution (25 mg/mL) in a 0.1 M phosphate buffer (pH = 7.0) was poured into a 0.05 M phosphate buffer at 37 °C through an 18 G plastic tube. The obtained atelocollagen thread was crosslinked with PegPRαCD1 or PluPRβCD1 via stepwise reductive amination in a 0.1 M borate buffer (pH = 8.5). The crosslinked thread was washed with an aqueous ethanol solution and dried under ambient conditions. The dry thread was fixed to the flexible polypropylene sheet using paper tape for a temporal fixation. Then, the liquid glue was applied to cover the part of the thread 5 mm from the paper tapes. After the liquid was solidified, the thread sample was subjected to tensile testing. 

### 2.6. Tensile Testing

A Micro Autograph MST-X HS/HR (SHIMADZU) was employed for the tensile testing. The fabricated thread samples were immersed in a 50 mM phosphate buffer (pH = 7) for 3 min before tensile testing under wet conditions. After the thread samples affixed to the polypropylene sheet were secured onto the measuring apparatus, the frame was severed, initiating the measurement process. The thread was pulled upward under a mist of water at the rate of 2 mm/min using the top jig connected to the 10 N load cell. The stress–strain curve of the sample was monitored in real time until the thread sample was broken. Statistical significance of the experimental data was assessed by Tukey’s test, where *p* < 0.01 and *p* < 0.05 were considered significant.

### 2.7. Analytical Methods and Apparatus

^1^H NMR measurement was performed on an ECS-400 spectrometer (JEOL). The powder PR sample (~5 mg) was dissolved in DMSO-d_6_ and employed for the 1D measurement at 293 or 318 K. The ^1^H NMR spectrum was obtained using 160 scans with a relaxation delay of 5 s.

Fourier-transform infrared (FT–IR) measurements of the synthesized PRs were performed using an IRAffinity-1S spectrometer (SHIMADZU). Powder PR samples were mixed and milled with potassium bromide (KBr), and the resulting powder mixture was fabricated into a pellet. The pellet was subjected to a transmittance measurement in the infrared region (4000–500 cm^−1^).

A TGA-50 thermogravimetric analyzer (SHIMADZU) was utilized to monitor the thermal decomposition of the samples during thermogravimetric analysis (TGA). The powder sample (~10 mg) was weighed on a platinum pan and placed in the sample chamber. The residual weight was monitored in real time while the sample was heated from 25 to 700 °C at the heating rate of 10 °C/min under N_2_ gas flow.

## 3. Results and Discussion

### 3.1. Synthesis and Characterization of PegPRαCD1

Peg_10k_PRαCD1 and Peg_20k_PRαCD1 were synthesized from the hydroxyl group-terminated Peg via a typical Gabriel amine synthesis process, *pseudo*-PR formation, terminal amide coupling by adamantane carboxylic acid, and catalytic oxidation of the primary alcohol groups of αCD (see Figure S1 for the adamantane end-capped PegPRαCD2). The aldehyde groups were selectively introduced as crosslinking sites into the four PRs via catalytic oxidation with TEMPO and PhI(OAc)_2_. Previous research used Dess–Martin Periodinane (DMP) to introduce the aldehyde groups into CDs of PRs, although the DMP potentially produces ketones besides aldehydes [24,25,26,27,28,29,30]. Most recently, we have reported that the TEMPO/PhI(OAc)_2_ redox couple is more suitable than the DMP for the selective oxidation of hydroxymethyl groups of CDs [23]. To calculate the number of crosslinking sites per PR molecule, aldehyde groups were pre-labeled with DMHZ via reductive amination. Previously, we reported the direct quantification of aldehyde groups as acetal forms by ^1^H NMR spectroscopy [23]. However, the limited solubility resulting from the increased molecular weight of PegPRαCD1 led to the development of an alternative method. The representative ^1^H NMR spectrum of PegPRαCD1-DMHZ is shown in Figure 2a. A broad spectrum is observed owing to the formation of PegPRαCD1, and its peaks are assigned to the αCD and Peg units. The N–CH_3_ protons (*k*) of DMHZ were detected at 2.54 ppm, while the N–H peak (*j*) likely overlapped with the peaks of the protons at the C_b,_ C_b’_, C_d_, and C_d’_ positions. The coverage ratio of 20 mol% was calculated from the ratio between the peaks obtained for the Peg units and the methyl groups. Moreover, the number of DMHZ molecules was consistent with that of αCD species, indicating that one aldehyde group was introduced per αCD molecule.

### 3.2. Synthesis and Characterization of PluPRβCD1

The *pseudo*-PR formation originates from the complexation between polypropylene glycol (Ppg) units and βCD [46], whereas βCD can move and rotate over the entire polymer units (i.e., Peg and Ppg units) [7,47]. To synthesize Plu_9k_PRβCD1 and Plu_15k_PRβCD1, we initially attempted to prepare the corresponding *pseudo*-PRs (i.e., PluPRβCD3) from Plu–COOH according to a previously developed procedure [46]. Although the earlier study reported the gram-scale preparation of *pseudo*-PR, the corresponding procedure was not reproduced under the same experimental conditions (i.e., a trace amount of *pseudo*-PR was obtained). Therefore, the preparation conditions were reinvestigated using Plu–OH as a model compound. One gram of Plu–OH was dissolved in a saturated aqueous solution of βCD (56 mL) and stirred at 25 °C for 7 days. A white suspension was obtained during stirring owing to the formation of *pseudo*-PR. The product was collected via centrifugation and freeze-dried. Further addition of βCD to the supernatant enabled the preparation of more *pseudo*-PR. The same procedure was applied for Plu–COOH. In total, approximately 0.7 g of PluPRβCD3 was obtained from the initial amount of Plu–COOH. The corresponding ^1^H NMR spectra were used to calculate the coverage ratio of βCD with respect to the total number of Peg and Ppg units, which was equal to 6 mol% (Figure S2). PluPRβCD2 was synthesized by the terminal end-capping of PluPRβCD3 via amide coupling with Trt–NH_2_ in anhydrous acetonitrile (Figure S3). Finally, one hydroxymethyl group per βCD in the PRs was converted to an aldehyde group using the TEMPO/PhI(OAc)_2_ redox couple. In addition to PegPRαCD1, the aldehyde groups of PluPRβCD1 were also successfully quantified using DMHZ (Figure 2b). The coverage ratio of 6 mol% was retained during terminal capping and catalytic oxidation.

### 3.3. FT–IR Measurements

**Figure 3:**
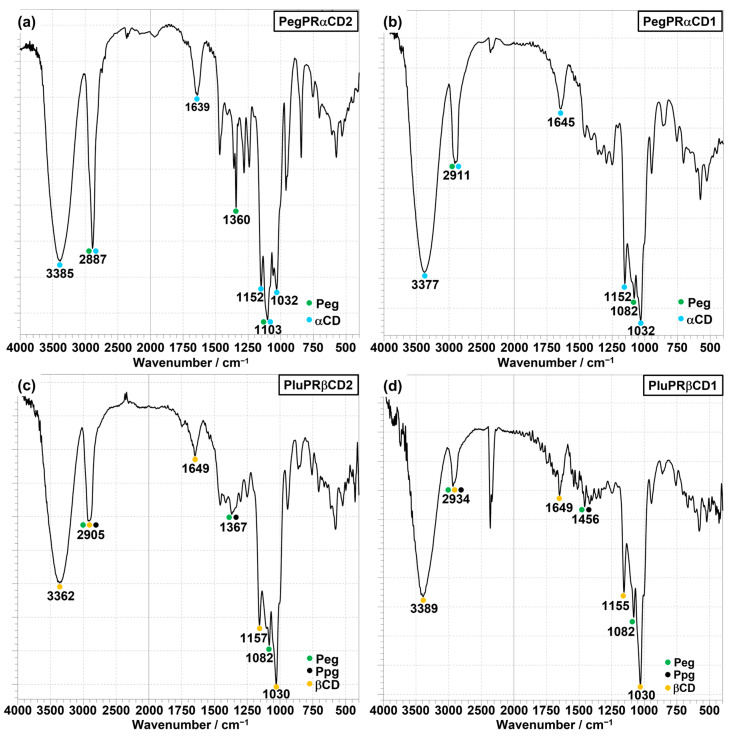
FT–IR spectra of (**a**) PegPRαCD2, (**b**) PegPRαCD1, (**c**) PluPRβCD2, and (**d**) PluPRβCD1.

Previous FT–IR studies produced peaks originating from Peg, Ppg, and CDs [48,49,50,51]. Comparative measurements with different coverage ratios enabled the assignment of characteristic peaks. Figure 3a shows a representative FT–IR spectrum of PegPRαCD2. The peaks at 1032 and 1152 cm^−1^ correspond to the C–O and C–O–C stretching vibrations of αCD, respectively. A new absorption band is observed at 1103 cm^−1^ because of the overlap between the αCD peaks and C–O stretching mode of Peg at 1082 cm^−1^ (Figure 3b–d). The peak at 2887 cm^−1^ is an overlap of the stretching vibrations of Peg C–H and free OH groups of αCD. In contrast, the wide absorption peak at 3385 cm^−1^ indicates the presence of the OH groups of αCD in the inter- and/or intramolecular hydrogen bonds. The bending vibration of αCD–C–H is observed at 1639 cm^−1^, while that of Peg is detected at 1360 cm^−1^. The PluPRβCD2 spectrum is similar to that of PegPRαCD2; however, its βCD peaks are relatively small owing to the lower coverage ratio (Figure 3c). The absorption peaks of Ppg almost completely overlap with those of the Pegs because of their similar vibration frequencies. The absorption peak of the stopper molecules (i.e., trityl groups) likely overlaps with that of the C–H bending vibration of βCD at 1649 cm^−1^. Interestingly, although aldehyde groups were successfully labeled with DMHZ for ^1^H NMR measurements, their characteristic peaks are absent from the FT–IR spectra (Figure 3b,d). Instead, the peaks originating from the presence of the free OH groups of CDs (located at 2911 and 2934 cm^−1^ in Figure 3b and Figure 3d, respectively) decreased after catalytic oxidation, confirming the production of acetal species from aldehyde and OH groups in the solid state [23]. Meanwhile, we cannot exclude the possibility of the hydration of aldehyde groups by residual water. The acetal formation also caused noticeable changes in CD absorption in the wavenumber range of 1250–1000 cm^−1^.

### 3.4. TGA Measurements

**Figure 4:**
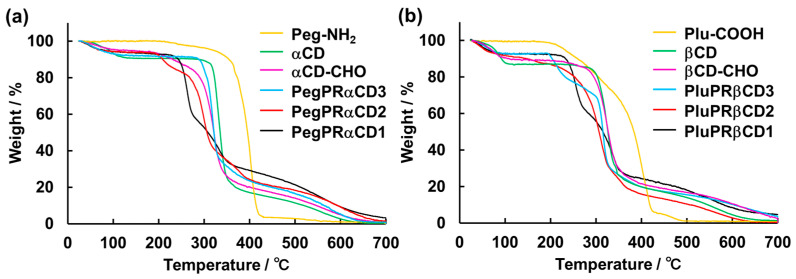
TGA curves: (**a**) Peg–NH_2_, αCD, αCD–CHO, PegPRαCD3, PegPRαCD2, and PegPRαCD1; (**b**) Plu–COOH, βCD, βCD–CHO, PluPRβCD3, PluPRβCD2, and PluPRβCD1. The samples were heated to 700 °C at a heating rate of 10 °C/min.

TGA was also performed to characterize PegPRαCD1 and PluPRβCD1 (Figure 4). The weight losses observed for Peg–NH_2_ are consistent with those of a previously reported TGA profile [52], thus validating the utilized experimental conditions (Figure 4a). αCD exhibited a weight loss due to the loss of moisture by 120 °C and decomposition of glucose units by 370 °C [53]. In comparison, the oxidation of one hydroxymethyl group of αCD did not cause a significant change in the TGA profile (i.e., αCD–CHO). The *pseudo*-PR PegPRαCD3 containing 20 mol% αCD produced weight losses arising from the thermal decomposition of both Peg–NH_2_ and αCD [50]. The weight loss of PegPRαCD2 was initiated at 200 °C owing to the decomposition of the stopper moiety [54], while its loss temperatures were lower than those of PegPRαCD3 above 320 °C. The weight loss of PegPRαCD1 started at 250 °C owing to the decomposition of polyacetal structures (i.e., inter- and/or intramolecular crosslinking) and hydrated aldehydes in the solid state [23]. In the temperature range above 350 ℃, the TGA curves of PegPRαCD2 and PegPRαCD1 are very similar. Meanwhile, Plu–COOH exhibits a clear two-step weight loss (Figure 4b). Because the significant thermal decomposition of Peg begins at around 350 °C (Figure 4a), the initial weight loss that starts at 200 °C results from the thermal decomposition of the Ppg units of Plu–COOH. The weight losses of βCD and βCD–CHO assigned to the loss of moisture (~110 °C) and decomposition of glucose units (~310 °C) were consistent with those observed in the previous studies [23,53]. Owing to the relatively low coverage ratio of PluPRβCD3, the decomposition of Ppg units after the loss of moisture is clearly observed between 200 and 310 °C. PluPRβCD2 exhibits a gradual decrease in the residual weight by 200 °C caused by the loss of moisture and thermal decomposition of trityl groups [55]. Similar to PegPRαCD1, the weight loss of PluPRβCD1, which begins at 250 °C, indicates the decomposition of polyacetal structures and Ppg units. However, unlike the other samples, no significant residual weight changes are observed for this compound in the temperature range above 350 °C. It is noteworthy that the introduction of aldehyde groups produced a similar trend in the TGA profiles, while the residual weights at the inflection points (i.e., 275 and 350 °C) strongly depended on the CD molar content.

### 3.5. Tensile Testing of PRATs

**Figure 5:**
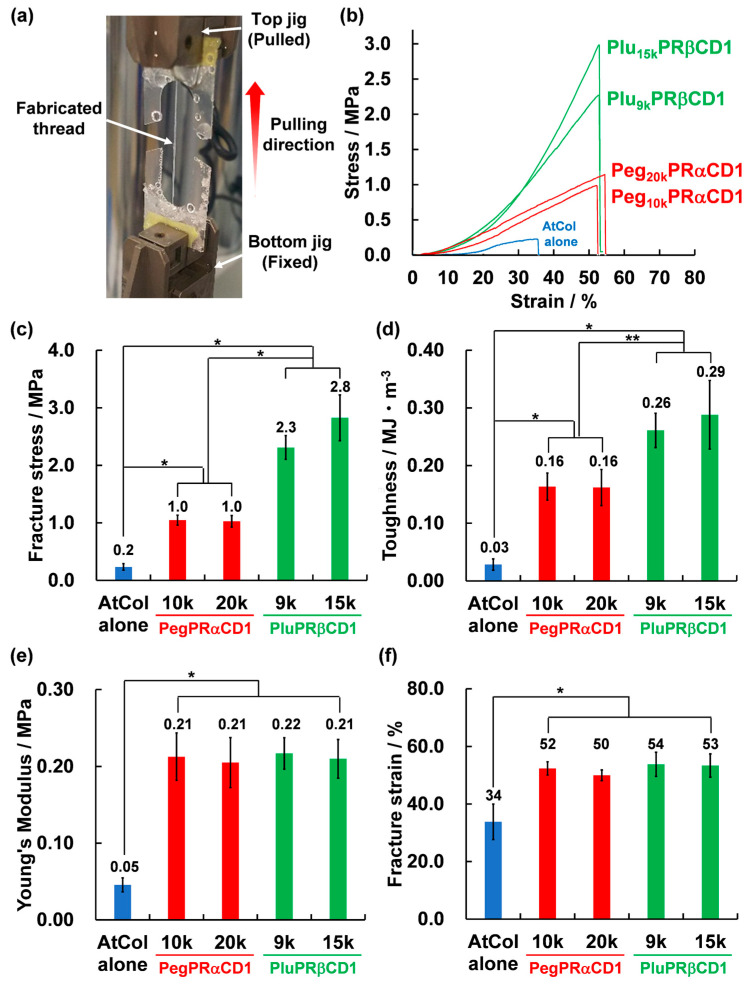
Tensile testing of AtCol alone and PRATs. (**a**) Representative photograph of the tensile testing setup. (**b**) Representative stress–strain curves. Bar graphs of the (**c**) fracture stress, (**d**) toughness, (**e**) Young’s modulus, and (**f**) fracture strain. Fracture stress was calculated by dividing the fracture test force (N) by the cross-sectional area (ca. 0.03 mm^2^). Young’s modulus was determined from the slope of the initial linear region (~6% strain) of the stress–strain curve. * *p* < 0.01, ** *p* < 0.05 (Tukey’s test).

The successful characterization of the synthesized polymers led to an investigation of the effect of PRs on the mechanical properties of the PRATs fabricated via reductive amination. The obtained threads contained wrinkles along the thread direction owing to the alignment of atelocollagen molecules [23], which were not previously observed for other types of soft materials based on collagen and atelocollagen (e.g., gels and membranes). Molecular alignment is potentially advantageous for increasing the strength and flexibility of PRATs. The results of tensile testing obtained under humidified conditions are presented in Figure 5 (Figure 5a shows a representative photograph of the utilized tensile testing setup). Crosslinking of atelocollagen thread (AtCol) by Peg_10k_PRαCD1 and Peg_20k_PRαCD1 increased both the fracture stress and strain as compared with AtCol alone (Figure 5b,c,f), leading to a 5-fold toughness increase (Figure 5d). The Young’s modulus calculated from the initial linear region of the stress–strain curve increased by a factor of 4 (Figure 5e). The obtained mechanical parameters reflected the entropic elasticity derived from the slide-ring characteristics of PegPRαCD1 [7,56]. However, the molecular weight of Peg did not significantly affect the mechanical properties of the synthesized PRATs. Previously, Takeoka et al. examined the extensibility of *N*-isopropylacrylamide-based polymer gels crosslinked by PRs with the same coverage ratio but different molecular weights of the axile polymer (i.e., Peg) [57]. Both the fracture stress and strain were enhanced by increasing the molecular weight of the PRs from 20,000 to 100,000, which indicated that the increased effective range of αCD resulting from the higher molecular weight significantly affected the gel mechanical properties. A similar effect of molecular weight was reported for atelocollagen hydrogels post-crosslinked with carboxymethyl PRs [58]. Note that molecular weight exerted a stronger effect on the fracture stress and strain than coverage ratio in the previous studies. The major difference between the PRATs and previously reported PR-crosslinked gels is the nature of matrix molecules. The matrix molecules were randomly oriented in the gels, whereas the atelocollagen molecules of the PRATs were tightly aligned along the thread direction [23]. Therefore, we tentatively concluded that the effects of the PRs depended on the alignment of the molecules to be crosslinked. We have found that Plu_9k_PRβCD1 and Plu_15k_PRβCD1 significantly enhance the mechanical properties of the PRATs. Plu_9k_PRβCD1 and Plu_15k_PRβCD1 further increased the fracture stress by approximately 2.3 times as compared with that of PegPRαCD1, maintaining the fracture strain (Figure 5b). Consequently, the toughness was further increased by a factor of 1.6 (no significant differences between Plu_9k_PRβCD1 and Plu_15k_PRβCD1 are observed in Figure 5c,d), while the Young’s modulus and fracture strain were maintained constant (Figure 5e,f). We found consistency in that molecular weight showed a negligible effect on the mechanical properties of the PRATs. In addition to the molecular alignment of atelocollagens, the observed effect of PluPRβCD1 was discussed based on the PR characteristics. One of the possible reasons for this phenomenon is the modified property nature of Peg units upon the application of an external force [59]. Ito et al. observed the crystallization of Peg units during the tensile testing of PR-crosslinked materials by lowering the coverage ratio (~2 mol%). Because PluPRβCD1 also contains wide uncovered Peg units, an external force applied to the PRATs may similarly induce the crystallization of Peg units. However, we cannot exclude the possibility that the ring size affects the slide-ring properties of the PRs. The increased ring size from αCD to βCD can promote solvent uptake into the CD inner cavity. The interactions between the OH groups of CDs, oxygen atoms in Peg units, and solvent molecules (i.e., water) facilitate the formation of hydrogen bonds, leading to molecular friction that prolongs the stress relaxation time [50,60]. These two factors, in addition to the alignment of atelocollagen molecules, may contribute to the unique mechanical properties of the produced PRATs. It should be noted that the increased fracture stress but maintained fracture strain provided by PluPRβCD1 had not been previously observed for PR-crosslinked materials. Further experimental investigations combined with theoretical studies regarding the unprecedented results should clarify the role of PRs as the characteristic components of the PRATs.

## 4. Conclusions

In this study, we investigated the effects of the molecular weight and coverage ratio of PRs on the mechanical properties of the produced PRATs. A novel method was established for the syntheses of PRs with relatively low coverage ratios. The obtained PRs were successfully characterized by ^1^H NMR, FT–IR, and TGA. Interestingly, the results of tensile testing demonstrated that coverage ratio was the main factor affecting the mechanical properties of the PRATs, whereas molecular weight produced a negligible effect on these properties. It is noteworthy that the effects of PRs on the PRAT properties potentially depend on the alignment of atelocollagen molecules. Although further investigations are required to support our conclusions and to further improve the mechanical properties for practical use in humans, the results of this study offer insights into the fundamental properties and applications of PR-reinforced biomaterials.

## Data Availability

Data sharing not applicable.

## Supplementary Materials

The following supporting information can be downloaded from: https://www.mdpi.com/article/10.3390/polym15153325/s1, ^1^H NMR spectra. Figure S1: ^1^H NMR spectrum of PegPRαCD2. Figure S2: ^1^H NMR spectrum of PluPRβCD3. Figure S3: ^1^H NMR spectrum of PluPRβCD2.
