# Supplementary material for: Synthesis, Characterization, and Potential Application of Cyclodextrin-Based Polyrotaxanes for Reinforced Atelocollagen Threads
Source: Polymers (Basel). 2023 Aug 7;15(15):3325. doi: 10.3390/polym15153325 (PMC10422439; doi:10.3390/polym15153325)
Supplement: Supplementary file 1 [file polymers-15-03325-s001.zip › polymers-2506640-supplementary.pdf]

Supplementary Materials
for

Synthesis, Characterization, and Potential Application of Cyclodextrin-Based Polyrotaxanes for Reinforced Atelocollagen Threads

Riku Kubota* and Ichiro Fujimoto

Koken Research Institute, Koken Co., Ltd., 1-18-36 Takarada, Tsuruoka-shi, Yamagata 997-0011, Japan

*Corresponding author

E-mail: riku.kubota@kokenmpc.co.jp

Tel.: +81-235-24-6251

^1H NMR spectra

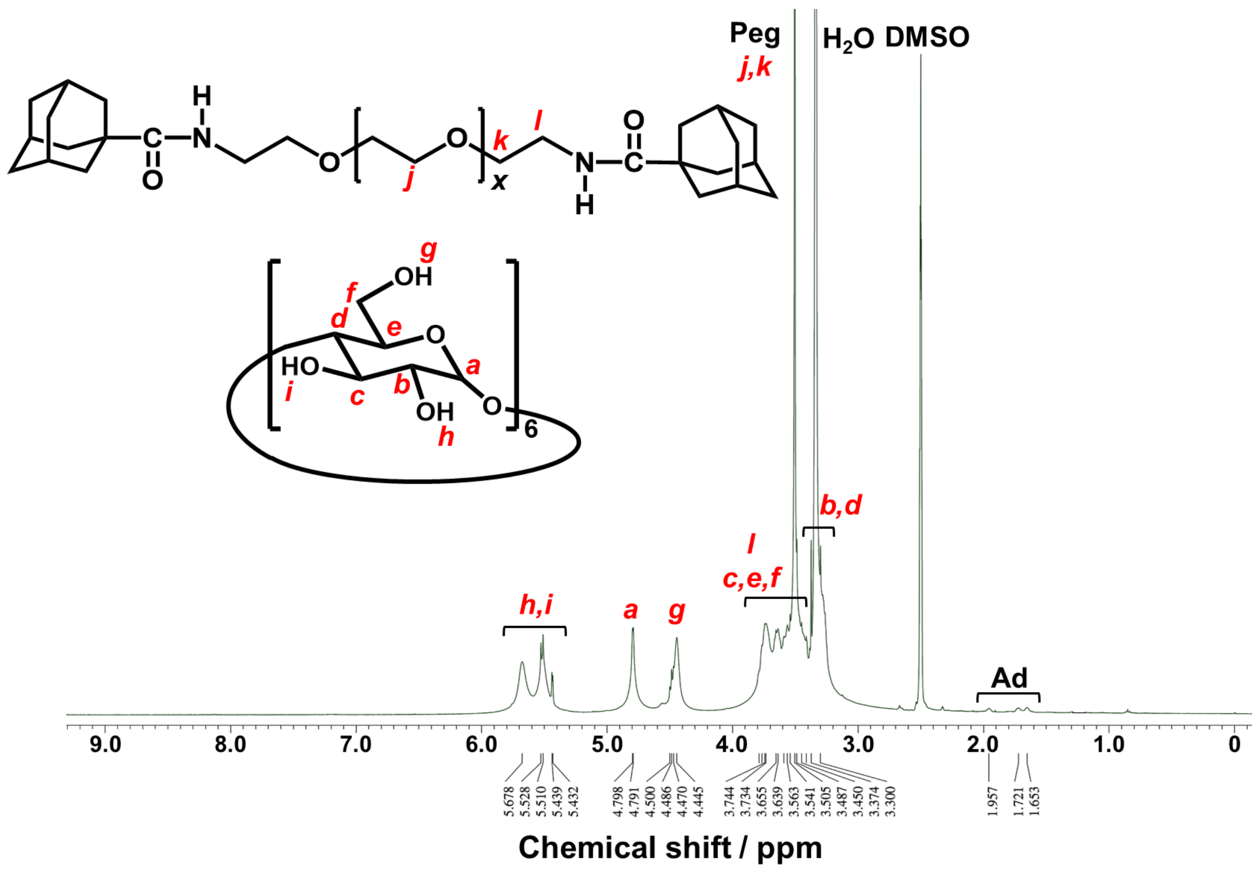

Figure S1. ^1H NMR spectrum of PegPRαCD2 (400 MHz, DMSO-d_6, 293K). Ad = adamantyl group

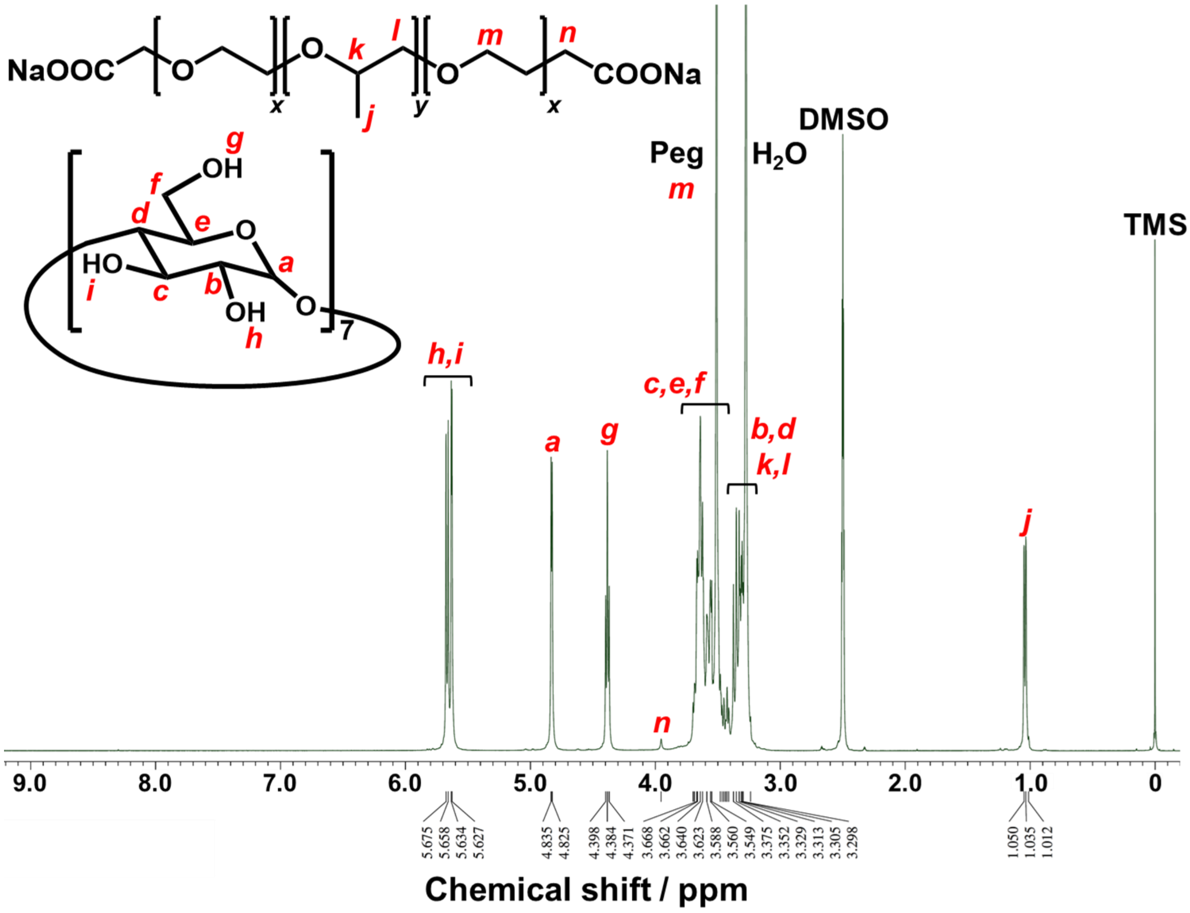

Figure S2. ^1H NMR spectrum of PluPRβCD3 (400 MHz, DMSO-d_6, 293K)

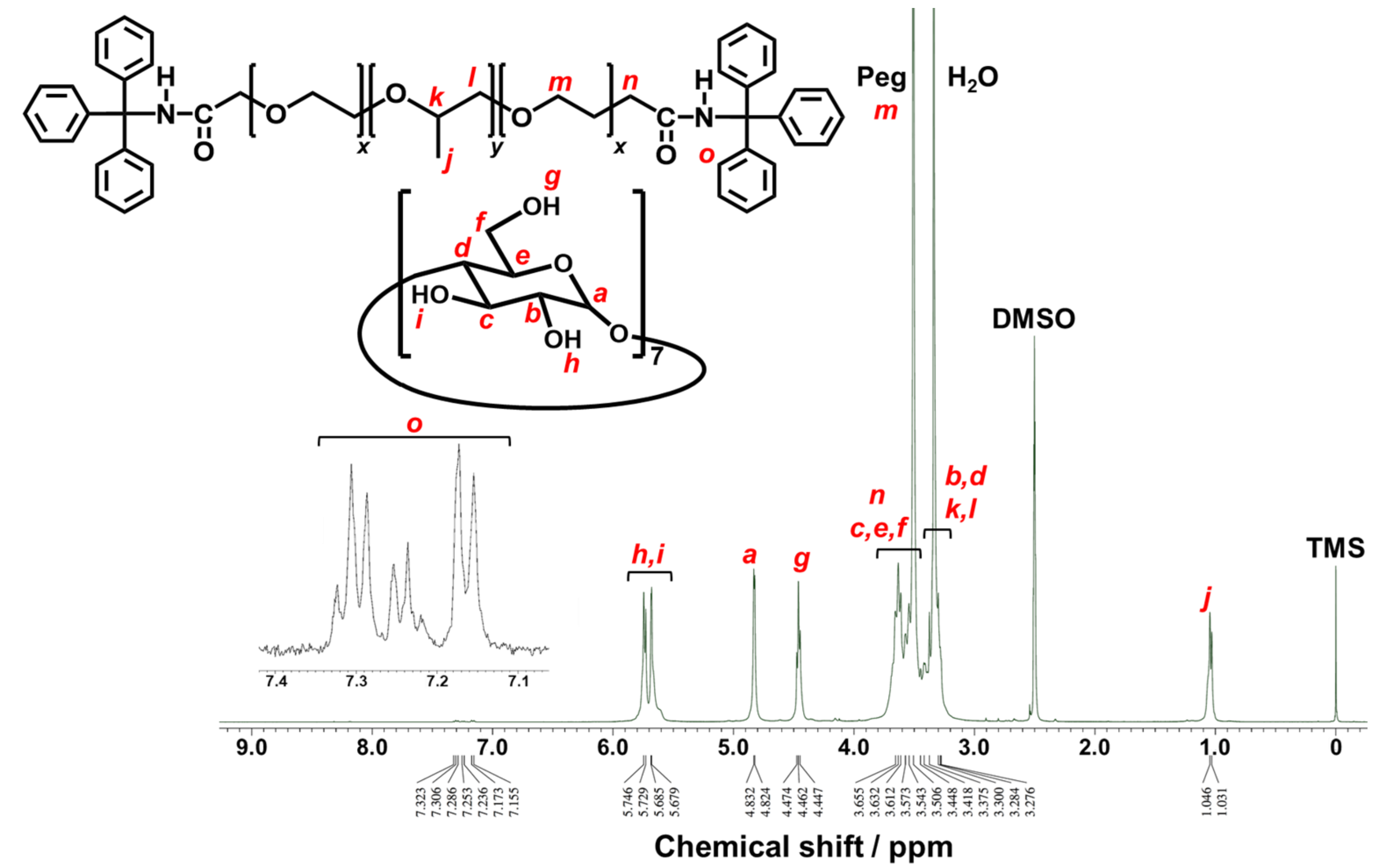

Figure S3. ^{1}H NMR spectrum of PluPRβCD2 (400 MHz, DMSO-d_6, 293K)
